# Digital scoring of EpCAM and slug expression as prognostic markers in head and neck squamous cell carcinomas

**DOI:** 10.1002/1878-0261.12886

**Published:** 2020-12-29

**Authors:** Henrik Schinke, Theresa Heider, Timm Herkommer, Florian Simon, Alexandra Blancke Soares, Gisela Kranz, Daniel Samaga, Laura Dajka, Annette Feuchtinger, Axel Walch, Laura Valeanu, Christoph Walz, Thomas Kirchner, Martin Canis, Philipp Baumeister, Claus Belka, Cornelius Maihöfer, Sebastian Marschner, Ulrike Pflugradt, Ute Ganswindt, Julia Hess, Horst Zitzelsberger, Olivier Gires

**Affiliations:** ^1^ Department of Otorhinolaryngology, Head and Neck Surgery Ludwig‐Maximilians‐University Munich Germany; ^2^ Research Unit Radiation Cytogenetics Helmholtz Zentrum München Neuherberg Germany; ^3^ Research Unit Analytical Pathology Helmholtz Zentrum München Neuherberg Germany; ^4^ Institute of Pathology Faculty of Medicine LMU Munich Germany; ^5^ Clinical Cooperation Group “Personalized Radiotherapy in Head and Neck Cancer“ Helmholtz Zentrum München Neuherberg Germany; ^6^ Department of Radiation Oncology Ludwig‐Maximilians‐University Munich Germany; ^7^ Department of Therapeutic Radiology and Oncology Medical University of Innsbruck Austria

**Keywords:** antigen scoring, EMT, EpCAM, HNSCC, Slug

## Abstract

Head and neck squamous cell carcinomas (HNSCCs) have poor clinical outcome owing to therapy resistance and frequent recurrences that are among others attributable to tumor cells in partial epithelial‐to‐mesenchymal transition (pEMT). We compared side‐by‐side software‐based and visual quantification of immunohistochemistry (IHC) staining of epithelial marker EpCAM and EMT regulator Slug in *n* = 102 primary HNSCC to assess optimal analysis protocols. IHC scores incorporated expression levels and percentages of positive cells. Digital and visual evaluation of membrane‐associated EpCAM yielded correlating scorings, whereas visual evaluation of nuclear Slug resulted in significantly higher overall scores. Multivariable Cox proportional hazard analysis defined the median EpCAM expression levels resulting from visual quantification as an independent prognostic factor of overall survival. Slug expression levels resulting from digital quantification were an independent prognostic factor of recurrence‐free survival, locoregional recurrence‐free survival, and disease‐specific survival. Hence, we propose to use visual assessment for the membrane‐associated EpCAM protein, whereas nuclear protein Slug assessment was more accurate following digital measurement.

## Graphical abstract

Epithelial‐to‐mesenchymal transition (EMT) impacts on the clinical outcome of head and neck squamous cell carcinomas (HNSCC). Here, we compared visual and digital scoring of epithelial marker EpCAM and EMT marker Slug in HNSCC for routine quantification of EMT. We propose to use visual assessment for membrane‐associated EpCAM, whereas nuclear protein Slug assessment was more accurate following digital measurement.

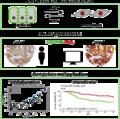

**Abbreviations**
DFS: disease‐free survival
DSS: disease‐specific survival
ECE: extracapsular extension
EGFR: epidermal growth factor receptor
EMT: epithelial‐to‐mesenchymal transition
EMT‐TF: epithelial‐to‐mesenchymal transition transcription factor
EpCAM: epithelial cell adhesion molecule
ERK1/2: extracellular‐regulated kinase 1/2
FFPE: formalin‐fixed paraffin‐embedded
Gy: gray
H&E: hematoxylin and eosin
HNSCC: head and neck squamous cell carcinoma
HPV: human papillomavirus
IFNγ: interferon‐gamma
IHC: immunohistochemistry
LMU: Ludwig‐Maximilians‐Universität
LR‐DFS: locoregional recurrence‐free survival
OCSCC: oral cavity squamous cell carcinoma
OS: overall survival
PD‐L1: programmed death ligand 1
pEMT: partial epithelial‐to‐mesenchymal transition
TCGA: The Cancer Genome Atlas
TMA: tissue microarray
UICC: Union for International Cancer Control

## 1 Introduction

Squamous cell carcinoma of the head and neck area (HNSCC) are frequent tumors that are associated with poor overall survival (OS) rates of 45% after 5 years [1]. Consistently, poor survival rates of HNSCC are connected to the presence of locoregional lymph node metastases at first diagnosis, to the existence of tumor cells that are resistant to radio‐(chemo)therapy, and to ensuing frequent local and regional recurrences. Next‐generation sequencing of primary HNSCC revealed a strong heterogeneity [2, 3, 4, 5] that correlated with poorer survival [6]. Bulk sequencing of primary HNSCC resulted in their classification into basal‐like, mesenchymal‐enriched, classical epithelial‐like, and atypical molecular subtypes [4, 7, 8]. Single‐cell RNA‐sequencing analysis of oral cavity carcinomas disclosed a need for a moderate revision of this classification and eliminated the mesenchymal subtype, which was the result of an enhanced presence of fibroblasts in the primary tumors. As a result, three subtypes were defined as malignant basal, classical, and atypical [9]. In addition to this reclassification, this study also disclosed single tumor cell signatures associated with cell cycle, cell stress, hypoxia, epithelial differentiation, and a partial epithelial/mesenchymal transition (pEMT), the latter one being associated with metastases formation. Interestingly, all malignant cells expressed epithelial markers, but a more pronounced retention of an epithelial phenotype was defined by the enhanced expression of epithelial cell adhesion molecule (EpCAM), keratins, and kallikreins. Epithelial differentiation and, thus, low levels of pEMT, is a positive prognostic marker. The value of EpCAM as a positive prognostic marker in HNSCC was confirmed independently by our group [10]. The analysis of EpCAM expression in combination with the expression of the epidermal growth factor receptor (EGFR), which is a therapeutic target in HNSCC, further disclosed that high expression of EGFR and low expression of EpCAM correlated with very poor survival. Oppositely, patients with EGFR^low^/EpCAM^high^ tumors were characterized by an outstandingly good prognosis [11]. EGFR has dual capacity to induce either proliferation or EMT in HNSCC, based on the degree of activation of the downstream effector extracellular‐regulated kinase 1/2 (ERK1/2). The association of EpCAM with a positive clinical outcome was functionally related to its ability to generate a novel ligand of EGFR through regulated intramembrane proteolysis of its ectodomain termed EpEX [11]. Cotreatment of HNSCC cell lines with EMT‐inducing concentrations of EGF and equimolar amounts of EpEX inhibited EGFR‐mediated EMT, along with reduced ERK1/2 phosphorylation and diminished transcription of EMT transcription factors (EMT‐TFs) Snail, Zeb1, and Slug [11]. Accordingly, the expression of phosphorylated ERK1/2 and Slug was enhanced in EGFR^high^/EpCAM^low^ HNSCC patients and correlated with reduced overall and disease‐free survival (OS, DFS) [11].

The epithelial signature of single HNSCC tumor cells was inversely correlated to a signature of pEMT, which was associated with an incomplete shift toward mesenchymal traits [9]. The pEMT signature was exclusively observed in the molecular subtype of basal‐like tumors in the small cohort analyzed and was associated with metastases formation, extracapsular extension, and lymphovascular invasion [9]. These findings were further confirmed in a larger cohort using results from the Cancer Genome Atlas (TCGA) of HNSCC. Hence, the authors concluded that pEMT is predominant in the basal‐like molecular subtype, and based on the association with metastases, they renamed this subtype into malignant basal [9]. Of note, the basal‐like molecular subtype of HNSCC was initially characterized by the overexpression of EGFR [7, 8], in its hyperphosphorylated, active form [7]. Unlike classical EMT observed during embryogenesis, where several EMT‐TFs are sequentially up‐regulated, pEMT in HNSCC was only accompanied by the enhanced expression of the EMT‐TF Slug in primary tumors, however, not at the level of single‐cell signatures [9]. Slug (also termed SNAI2) is a zinc finger transcription factor of the C2H2 (cysteine/histidine) family that primarily acts as a repressor of gene expression, including the cell adhesion molecule E‐cadherin [12] and pro‐apoptotic genes [13, 14]. Expression of the EMT‐TF Slug is considered an early event in the induction of EMT [15], which is commonly followed by the further induction of additional EMT‐TFs such as Zeb1.

Recently, the expression of four genes of the pEMT signature defined by Puram *et al*. was assessed in a cohort of oral cavity squamous cell carcinomas (OCSCCs). The marker of epithelial differentiation SPRR1B and three markers of pEMT (LAMC2, LAMB3, PDPN) were assessed in OCSCC in the absence or presence of lymph node metastases to determine their prognostic value. pEMT markers were associated with higher grades, perineural invasion, and node positivity, but did show only a tendency to correlate with poorer overall and disease‐free survival (OS and DFS) [16]. Hence, a partial shift to mesenchymal features contributes to the heterogeneity of HNSCC and associates with unfavorable disease outcome and metastases formation. Quantification of pEMT in HNSCC could thus provide a valuable criterium to stratify patients beyond TNM classification and human papillomavirus (HPV) status. In the present study, we have compared visual and digital software‐based analyses of the expression of the epithelial marker EpCAM and the pEMT‐associated EMT‐TF Slug in primary HNSCC. Similar comparative assessments of both evaluation techniques have been performed for the radiation biomarker CLIP2 in pediatric papillary thyroid carcinomas [17]. Visual analysis relies on the quantification of the antigen expression with respect to intensity and frequency in tumor areas by experienced experimenters. Typically, scoring systems implement the staining intensity from 0 to 3 represent no, weak, moderate, and strong antigen expression, and the proportion of antigen‐expressing tumor cells. The latter is usually provided as proportion ranges [18] or as estimated percentages [10]. Digital software‐based analyses compute antigen expression based on algorithms in areas of interest that are predefined by experimenters [19]. Particular interest has been set on the quantification of the Her2 antigen in the frame of Herceptin‐based treatment of breast cancer patients and digital evaluation may improve consistency and accuracy of quantification [20].

Here, we demonstrate that a robust assessment of EpCAM can be performed with visual and digital analyses, whereas the expression of Slug might be overestimated following visual assessment. In summary, software‐based evaluation of EpCAM and Slug is a valid tool to prognosticate the clinical outcome of HNSCC patients.

## 2 Materials and methods

### 2.1 Human biospecimen

Biospecimen is reported according to REMARK standards [21]. The retrospective “LMU‐KKG” cohort analyzed in the present study is composed of primary tumor samples of *n* = 102 patients with HNSCC [22, 23]. Tumor stage was assessed using the UICC TNM Classification of Malignant Tumors, 7th edition. HPV status of the patients was determined by p16^INK4a^ immunohistochemistry in combination with HPV DNA detection as described before [24]. Age, sex, tumor localization, TNM, perineural invasion, extracapsular extension, grading, UICC staging, and HPV status of all patients are provided in Table 1. Oropharyngeal squamous carcinomas (47%) and oral cavity carcinomas (28%) represented the most prevalent tumors. All patients received surgical resection followed by adjuvant radiotherapy at the LMU Department of Radiation Oncology between 2008 and 2013 with a median dose of 64 Gy (median dose 2 Gy per fraction). In the case of close or positive microscopic resection margins and/or ECE, patients (*n* = 67) received concurrent chemotherapy. The majority (73%) of the patients received CDDP/5‐fluorouracil (5‐FU; CDDP: 20 mg·mÇ^−1^ BSA day 1–5/29–33; 5‐FU: 600 mg·mÇ^−1^ BSA day 1–5/29–33). In selected cases, mitomycin C (MMC) or 5‐FU/MMC replaced platin‐based chemotherapy.

**Table 1:** Clinical parameters of HNSCC cohort including sex, age, localization, TNM classification, perineural invasion status, extracapsular extension status, grade, and UICC stage. Nd: not determined.

Variable	Number of patients (total *n* = 102)	Percentage
Sex		
Male	72	70.6%
Female	30	29.4%
Median age (range)	61 years (20–84)	
Localization		
Hypopharynx	15	14.7%
Larynx	11	10.8%
Oral cavity	28	27.5%
Oropharynx	48	47%
T classification		
T1	15	14.7%
T2	41	40.2%
T3	30	29.4%
T4	16	15.7%
N classification		
N0	30	29.4%
N1	24	23.5%
N2a	5	4.9%
N2b	27	26.4%
N2c	14	13.7%
N3	2	2%
M classification		
M0	102	100%
M1	0	0%
Perineural invasion		
Pn0	53	52%
Pn1	19	18.6%
*nd*	30	29.4%
Extension		
ECE^−^	43	60.5%
ECE^+^	28	39.5%
Grade		
I	3	2.95%
II	38	37.25%
III	61	59.8%
UICC stage		
I	1	0.98%
II	9	8.8%
III	32	31.4%
Iva	57	55.9%
IVb	3	2.95%
HPV status		
HPV^neg^	80	78.4%
HPV^pos^	22	21.6%

Formalin‐fixed paraffin‐embedded (FFPE) tumor specimens were derived from surgically resected tumor tissue samples. For the tissue microarray (TMA), all available hematoxylin and eosin (HE)‐stained FFPE tumor sections were microscoped and a representative tissue block was selected. All tumor specimens were histologically re‐evaluated by a pathologist (A.W.) and the tumor area defined. From each tumor specimen, three representative tissue cores (1 mm diameter) taken from different regions were arrayed. 3‐µm sections of the TMAs were HE‐stained, and sequential 3‐µm sections were used for immunohistochemical staining.

### 2.2 Immunohistological staining

IHC staining was performed under standardized conditions on a Discovery XT digital stainer (Ventana Medical Systems, Roche Diagnostics, Penzberg, Germany) using monoclonal antibodies directed against human EpCAM (#2929, VU1D9, anti‐mouse, Cell Signaling Technology, Danvers, MA, USA) and SLUG (#9585, C19G7, anti‐rabbit, Cell Signaling). Slides were incubated with primary antibodies (rat monoclonal anti‐SLUG (1 : 250), mouse monoclonal anti‐EPCAM (1 : 250)) in Dako REAL antibody dilution (Agilent Technologies, Santa Clara, CA, USA), incubated with respective secondary antibodies (anti‐mouse and anti‐rabbit, ready‐to‐use universal secondary antibody, 760‐4205, Ventana Medical Systems, Roche Diagnostics), and detected by the Discovery DAB Map Kit (Ventana Medical Systems, Roche Diagnostics). For each staining procedure, additional respective positive and negative controls were included.

### 2.3 Digital scoring

Following H&E staining and IHC, tissue microarray slides were scanned at ×20 original objective magnification using a digital Mirax Desk slide scanner (Carl Zeiss Microscopy GmbH) before importing into the image analysis software Definiens Developer XD2 (Definiens AG), as described previously [19]. This software allows to detect and quantify the immunohistochemical staining intensities in different cellular compartments (e.g., membranes) within a user‐specified region of interest of the tumor cells. In brief, tumor areas containing at least 70% tumor cells were visually annotated, and a rule set for each antigen marker was defined by operators to detect and quantify morphometry data such as color and shape parameters of nucleus and cytoplasm from H&E staining, and staining intensities from IHC in the annotated tumor area. The algorithms were adjusted blinded with regard to corresponding clinical outcomes as to detect nuclear SLUG expression and cytoplasmic/membranous EpCAM expression. The quantified parameter for EpCAM and SLUG was a value representing a point on a continuous spectrum of average brown intensity in relative units.

### 2.4 Visual Scoring

Immunohistochemistry intensity scores (IHC score) were generated for each sample within the cohort as the product of antigen expression intensity (0–3) and the percentage of expressing tumor cells within biospecimens, as described [10, 25]. IHC scores are averages of values independently assessed by two experimenters, who were blinded to clinical staging and outcome of patients. EpCAM and Slug expression was analyzed separately for each individual antigen in order to preclude bias with respect to correlation of antigen expression.

### 2.5 Clinical endpoints and survival analysis

Overall survival (OS), recurrence‐free survival (RFS), locoregional recurrence‐free survival (LR‐RFS), and disease‐specific survival (DSS) served as clinical endpoints. OS was calculated in months from the date of radiotherapy treatment start to the date of death due to any cause. RFS was calculated in months from the date of radiotherapy treatment start point to the first observation of any recurrence or death. LR‐RFS was calculated in months from the date of radiotherapy treatment start point to the first observation of locoregional recurrence or death. DSS was calculated in months from the date of radiotherapy treatment start point to the date of HNSCC‐related death. In the absence of an event, patients were censored at the date of the last follow‐up visit.

Data analysis was performed using R Core Team, R: A Language and Environment for Statistical Computing, R Foundation for Statistical Computing, 2017; r version 3.6.1 (2019‐07‐05); and the R‐survival package (CRAN). For univariable analysis, IHC expression scores of EpCAM and Slug resulting from visual and digital quantification were included into Cox proportional hazard models. If resulting Cox proportional hazard models and variables were significant according to log‐rank and Wald statistic *P*‐value, patient cohorts were dichotomized into low and high expressors according to median values of IHC expression scores. Dichotomized cohorts were implemented in Kaplan–Meier plots with median survival times, Cox proportional hazard models’ hazard ratios, 95% confidence interval ratios, and log‐rank *P*‐values. For multivariable analysis, univariable analyses of OS, RFS, LR‐RFS, and DSS for the variables T‐status (tumor size), N‐status (lymph node metastases), R‐status (resection), Pn‐status (perineural invasion), grading, ECE (extracapsular extension), HPV status, and EpCAM and Slug quantification visual and digital were conducted. For each clinical endpoint, significant variables according to Wald statistic *P*‐values were implemented into multivariable Cox proportional hazard models.

Additional statistical analysis was performed using r with the r stats package and the built‐in functions from the tidyverse package (CRAN).

## 3 Results

### 3.1 Cohort description

In order to address potential differences in the evaluation of the expression and the prognostic value of EpCAM and Slug between visual and digital assessment, a tissue microarray (TMA) from the retrospective LMU‐KKG (Ludwig‐Maximilians‐University of Munich, Clinical Cooperation Group “Personalized Radiotherapy in Head and Neck Cancer) cohort of *n* = 102 HNSCC primary tumors including patients who had undergone surgical resection followed by adjuvant radio(chemo)therapy was generated. The cohort parameters are summarized in Table 1. In short, the cohort comprises mainly patients with squamous cell carcinoma of the head and neck in advanced stages (87.3% UICC III/IV), including 21.6% HPV‐positive tumors.

### 3.2 Visual and digital assessment of EpCAM and Slug expression

**Fig. 1:**
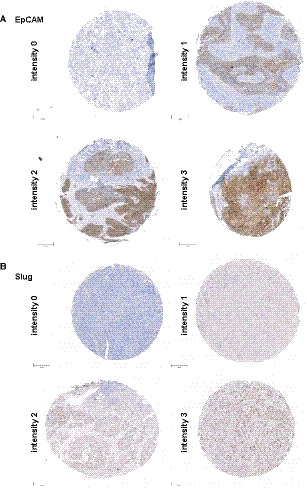
EpCAM and Slug expression intensities in HNSCC tissue microarrays. Shown are representative examples of EpCAM (A) and Slug (B) staining in tissue microarrays with the different staining intensities ranging from no (0), weak (1), intermediate (2), to high expression (3). Antigen staining is visualized in brown; counterstaining with hematoxylin/eosin is visualized in blue. Scale bars represent 200 µm.

TMA was stained with EpCAM‐ and Slug‐specific antibodies and was evaluated in a blinded manner by two independent reviewers. The samples were classified as antigen‐negative (0), or as weakly (1), intermediately (2), or strongly antigen‐positive (see representative examples of each expression strength in Fig. 1). For the visual evaluation, an established immunohistochemistry scoring (IHC score) composed by the multiplication of the staining intensity (0–3) and the percentage of cells at each staining intensity resulting in a maximal score of 300 were used as reported [25]. Visual assessment of EpCAM expression yielded an IHC score range of 0–218, median of 26.7, mean of 60.8, and 1^st^ and 3^rd^ quartile of 0 and 116.3, respectively. Visual assessment of Slug expression yielded an IHC score range of 0–280, median of 60, mean of 71.5, and 1^st^ and 3^rd^ quartile of 10 and 110, respectively (Table 2).

**Table 2:** Comparison of visual and digital scoring of EpCAM and Slug expression in HNSCC patients (*n* = 102). Score range 0–300.

Variable	EpCAM		Slug	
	Visual	Digital	Visual	Digital
Range (Min‐Max)	0–218	0–223	0–280	0.27–121
Median	26.7	50.7	60	28.7
Mean	60.75	61	71.5	33.7
1st quartile	0	28.5	10	10.7
3rd quartile	116.25	88	110	48.5

**Fig. 2:**
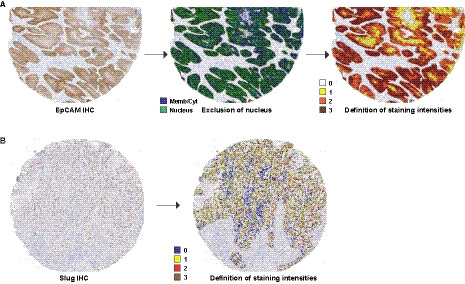
Software‐based determination of EpCAM and Slug expression intensities in HNSCC tissue microarrays. (A) Tissue microarray samples stained for the expression of EpCAM were analyzed with the Definiens Developer XD2 (Definiens AG) to identify nuclei and the remaining membrane and cytoplasmic areas (middle panel). EpCAM staining intensity was thereafter determined in the membrane/cytoplasm as no (0, white), weak (1, yellow), intermediate (2, orange), to high expression (3, brown). (B) Tissue microarray samples stained for the expression of Slug were analyzed with the Definiens Developer XD2 (Definiens AG) to identify nuclei and Slug staining intensities were determined as (0, blue), weak (1, yellow), intermediate (2, red), to high expression (3, brown).

Next, the Definiens Developer XD2 (Definiens AG) served to automate EpCAM and Slug expression assessment following immunohistological staining. Based on the differential localization of EpCAM and Slug, different protocols were used for the identification of antigen staining. EpCAM is a transmembrane protein that is primarily expressed at the plasma membrane and can further be detected in the cytoplasm, owing to endocytosis [26]. Therefore, recognition of the cell membrane and the cytoplasm was optimized in order to exclude the nucleus for the assessment of EpCAM expression (Fig. 2A, middle panel). Thereafter, EpCAM expression was determined in the plasma membranous and cytoplasmic area as antigen‐negative (0), or as weakly (1), intermediately (2), or strongly antigen‐positive (see representative examples of each expression strength in Fig. 2A, right panel). The assessment of the nuclear protein Slug was quantified exclusively in the nucleus, as antigen‐negative (0), or as weakly (1), intermediately (2), or strongly antigen‐positive (see representative examples of each expression strength in Fig. 2B, right panel). Digital assessment of EpCAM expression yielded an IHC score range of 0–223, median of 50.7, mean of 61.0, and 1^st^ and 3^rd^ quartile of 28.5 and 88.0, respectively. Digital assessment of Slug expression yielded generally lower expression scores compared to visual scoring, with an IHC score range of 0.27–121, median of 28.7, mean of 33.7, and 1^st^ and 3^rd^ quartile of 10.7 and 48.5, respectively (Table 2). Hence, the digital assessment yielded similar values for EpCAM, but disclosed reduced values for the expression of Slug compared to visual scoring.

### 3.3 Comparison of digital and visual assessment of EpCAM and Slug expression in HNSCC

**Fig. 3:**
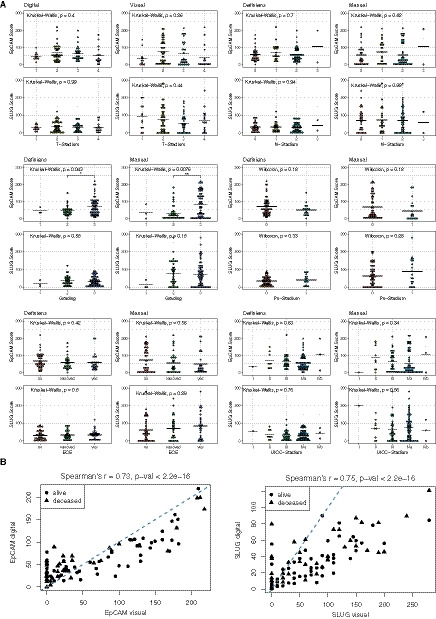
Correlation of EpCAM and Slug expression levels with clinical parameters of HNSCC patients. (A) Digital and visual quantification of EpCAM (upper panels) and Slug (lower panels) expression levels was classified according to the T‐ and N‐status, grading, perineural invasion (Pn‐status), extracapsular extension (ECE), and the UICC‐status (Union International Contre le Cancer). EpCAM and Slug expression scores (0–300) for all *n* = 96/102 HNSCC patients are presented as jitter plots with mean values (line) and *P*‐values from Kruskal–Wallis tests (group size > 2) or Wilcoxon tests (group size = 2). (B) IHC scores from the digital and visual quantification of EpCAM and Slug are presented a percentage of the maximal scores. Shown are correlation curves with rho (*r*) and *P*‐values from Spearman´s rank correlation analyses. Patients’ survival or death is depicted squares and circles, respectively. Specific *P*‐values are indicated.

EpCAM and Slug expression intensities resulting from digital and visual scoring were tested for independence of the clinical parameters tumor size (T), lymph node involvement (N), grading, PNI, ECE, and UICC staging for a total of *n* = 96 patients for whom all parameters were available by Kruskal–Wallis or Wilcoxon statistical testing. Moderately increased mean expression levels of EpCAM were significantly associated with poorly versus moderately differentiated tumors following digital and visual evaluation (Fig. 3A). No significant associations were observed between EpCAM and Slug expression and all other clinical parameters (Fig. 3A).

In order to further compare digital and visual scoring, a Spearman correlation analysis across all patients was performed. Digital and visual EpCAM expression scores for each patient correlated positively with a value of *r* = 0.73 and a *P*‐value < 2.2e‐16 (Fig. 3B). Digital and visual Slug expression scores for each patient also correlated positively with a value of *r* = 0.75 and a *P*‐value < 2.2e‐16 (Fig. 3B). Additionally, the survival status of each patient is depicted in the correlation curves. Next, absolute values for EpCAM and Slug scoring resulting from digital and visual scoring were compared across all patients. Mean scores of EpCAM expression did not show any difference between digital and visual evaluation (Fig. S1). Paired comparisons at the single patient level showed some degree of variation (see connecting line in Fig. S1). Unlike EpCAM, mean scores of Slug expression did vary significantly between digital and visual scoring, with the latter one resulting in higher scores (Fig. S1).

### 3.4 Digital and visual assessment of EpCAM and Slug expression in correlation with clinical parameters and endpoints

**Fig. 4:**
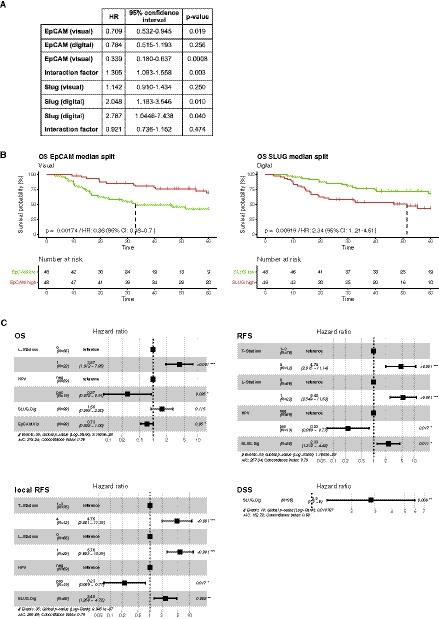
Stratification of HNSCC patients according to EpCAM and Slug expression for clinical endpoints. (A) Hazard ratios (HR), 95% confidence intervals, and *P*‐values are indicated for each antigen and evaluation technique per 50 units of IHC score. (B) Stratification of HNSCC patients according to median expression over a time period of 5 years (time is given in months) is shown as Kaplan–Meier curves for with HR, 95% CI, *P*‐values, and numbers of patients at risk in each risk group over time (antigen high and low, red and green color, respectively). (C) Multivariable analyses of overall survival (OS), recurrence‐free survival (RFS), locoregional recurrence‐free survival (LR‐RFS), and disease‐specific survival (DSS). Shown are Forest plots from multivariable Cox proportional hazard models including all variables shown to have prognostic significance in univariable analyses for each clinical endpoint with HRs, 95% CIs, events, Akaike information criterion (AIC), Concordance index, global log‐rank *P*‐values, and variable‐specific *P*‐values. Dig, digital scoring; HPV, human papillomavirus; L‐status, lymph vessel invasion; T‐status, tumor size; Vis, visual scoring. HRs and CIs are indicated for each antigen and evaluation technique per 50 units of IHC score.

Continuous Cox proportional hazard models (CoxPH) were computed to compare the prognostic power of EpCAM and Slug quantification in dependency of the assessment methods with respect to the OS of HNSCC patients, as a central clinical endpoint (Fig. 4A). Visual assessment of EpCAM and digital assessment of Slug allowed to stratify HNSCC patients according to the median antigen expression within our cohort with respect to OS (EpCAM‐visual: HR 0.36; 95% CI: 0.18–0.70; *P*‐value = 0.00174; Slug‐digital: HR 2.34; 95% CI: 1.21–4.51; *P*‐value = 0.00919; Fig. 4B). Refinement of the model based on digital assessment of Slug with the interaction factor to the visual assessment did not improve the initial CoxPH. In contrast, refinement of the CoxPH model based on visual assessment of EpCAM with the interaction factor improved the initial CoxPH model (Fig. 4A).

Univariable Cox proportional hazard models were computed with respect to OS, recurrence‐free survival (RFS), locoregional RFS (LR‐RFS), and disease‐specific survival (DSS) for the variables tumor size and nodal metastases (T‐ and N‐status), resection margin status (R‐status), perineural invasion (Pn‐status), lymphovascular invasion (L‐status), grading, extracapsular extension (ECE), HPV, EpCAM, and Slug levels, both following visual and digital quantification (Fig. S2). Variables that were significantly associated with OS (L‐status, HPV, Slug‐digital, EpCAM visual), RFS (T‐status, L‐status, HPV, Slug‐digital), LR‐RFS (T‐status, L‐status, HPV, Slug‐digital), and DSS (Slug‐digital) in univariable analyses were implemented into multivariable Cox proportional hazard models. Multivariable analysis determined a positive L‐status as independent negative prognostic marker of OS and HPV positivity and high EpCAM expression quantified by visual means as independent positive prognostic markers of OS. T‐status, L‐status, and high expression of Slug quantified by digital means were negative prognostic markers, and HPV positivity was a positive prognostic marker of RFS and LR‐RFS. Interestingly, high expression of Slug quantified by digital means was the only negative prognostic marker of DSS (Fig. 4C).

## 4 Discussion

HNSCC is associated with considerable inter‐ and intratumoral heterogeneity resulting from a high yield of DNA mutations [4, 5], with HPV‐driven HNSCC displaying fewer and different mutations than classical HNSCC [27]. Next‐generation sequencing confirmed a high degree of heterogeneity at the transcriptional level and revealed the existence of basal‐like, mesenchymal‐enriched, classical epithelial‐like, and atypical molecular subtypes [4, 7, 8]. Based on single‐cell RNA sequencing of oral cavity cancers, molecular subtypes were refined to malignant basal, classical, and atypical subtypes [9]. The malignant basal subtype comprises tumors with a high expression of EGFR, frequently in its activated phosphorylated form, that have undergone a partial EMT [7, 8, 9]. Hyperactivation of EGFR in HNSCC cells induces EMT through the extracellular‐regulated kinases ERK1/2, which results in the enhanced expression of EMT‐TFs including Slug, Snail, and Zeb1 [11]. Furthermore, EGFR expression and activating phosphorylation were observed in a subcluster of HNSCCs that was defined by a low cytotoxic immune phenotype and reduced expression of programmed death ligand 1 (PD‐L1) and interferon‐gamma (IFNγ) [28]. Hence, EMT in general and more specifically EGFR‐mediated EMT is involved in the regulation of metastasis formation and therapy resistance and might potentially shape interactions with immune cells.

In light of the growing importance of cellular changes related to EMT in HNSCC and other carcinoma types [29, 30, 31], we have performed a technical comparison of manual and optical scoring systems for the central epithelial marker EpCAM and the EMT‐TF Slug in HNSCC. Unlike the frequently used semiquantitative visual evaluation, software‐based quantification of EpCAM and Slug allowed to predefine cellular areas of interest and thereby to standardize procedures. We chose to quantify EpCAM at the plasma membrane and in the cytoplasm, whereas staining of the nuclear TF Slug was considered exclusively in the nucleus (Fig. 2). Furthermore, staining intensities were predefined and were equally applied to all samples, thus minimizing a potential interinvestigator bias and some degree of subjectivity. It is also important to note that FFPE samples were used in a TMA format that is feasible for routine diagnostic protocols. The cohort studied in this comparative work represents a cross section of HNSCC patients with a majority of male patients, higher frequency of oral cavity and oropharynx carcinomas, an even distribution of small (T1/2) and larger tumors (T3/4), and 70% of patients with lymph node metastases at initial diagnosis (Table 1).

A comparison of digital and visual antigen quantification revealed that results of EpCAM expression did not vary considerably between both techniques. IHC score ranges, median, mean, 1^st^ and 3^rd^ quartile of antigen expression did differ neither at the level of the entire cohort nor substantially at the single patient level (Table 2, Fig. 3A,B). Furthermore, a statistical evaluation of potential differences of EpCAM quantification methods in association with the clinical parameters TNM status, perineural invasion, extracapsular invasion, grade, and UICC stage did not reveal any significant differences, except between grades 2 and 3 (Fig. 3A). Hence, these results suggest that both techniques are equally feasible for the quantification of EpCAM in HNSCC samples. Comparable results have been reported for the quantification of the therapeutic target Her2 in breast cancer patients with a high congruence of visual and digital assessments [32, 33, 34]. Based on the knowledge that EpCAM is a positive prognostic marker whereas Slug is a negative prognostic marker in HNSCC [9, 10, 35], univariable Cox proportional hazard models were generated. Despite similar quantification results for EpCAM, only visual assessment revealed a significant prognostic power, which could be improved by refinement with the interaction factor computed from both assessment methods (Fig. 4A,B).

Although of interest with respect to the complex biology of EpCAM [36], cleavage and nuclear translocation of its intracellular domain [37] was not quantified in the present study for several reasons. From our experience, nuclear staining of EpICD is not homogeneous but presents as a speckled pattern with intercellular differences [37]. Nuclear translocation of EpICD depends upon the degree of regulated intramembrane proteolysis, which affects a variable proportion rather than the totality of full‐length EpCAM molecules and can differ within tumor areas. Furthermore, EpICD is a labile protein moiety that is subject to efficient degradation by the proteasome complex [37, 38]. Owing to its labile nature and to the differential localization of EpICD in the context of full‐length EpCAM and following cleavage, technical hurdles have to be overcome when considering antigen‐retrieval protocols in FFPE samples. These aspects might account for differences in EpICD staining observed between native cryo‐conserved specimens as well as cell lines [26, 37] and FFPE samples [39, 40], despite using identical antibodies.

Unlike EpCAM, digital and visual quantification of Slug in HNSCC samples differed substantially (Fig. 3B, Fig. S1). Range, median, mean, and 3^rd^ quartile of Slug expression were higher following visual quantification (Table 2). Thus, visual evaluation of the nuclear antigen Slug can result in an overrating of the expression frequency and strength compared with digital quantification, which might be attributable to subjective color perception [19]. Analyzing Slug quantification of both methods for independence of clinical parameters revealed no significant differences overall (Fig. 3A). Interestingly, only digital assessment allowed to generate a univariable CoxPH model predicting the patients´ OS (Fig. 4A,B).

Multivariable analyses were performed to gain further insight in the performance of EpCAM and Slug quantification methods compared to established markers for the clinical endpoints OS, recurrence‐free survival (RFS), locoregional RFS, and disease‐specific survival (DSS). Tumor size and nodal metastases (T‐ and N‐status), resection margin status (R‐status), perineural invasion (Pn‐status), lymphovascular invasion (L‐status), grading, extracapsular extension (ECE), HPV status, EpCAM, and Slug levels, both following visual and digital quantification, were implemented in these analyses (univariable CoxPH models in Fig. S2). Here, visual quantification of EpCAM was an independent positive prognostic marker of OS, whereas digital quantification of Slug was an independent negative prognostic marker for RFS, LR‐RFS, and the only marker for DSS (Fig. 4C). Thus, within the presented cohort, EpCAM‐visual and Slug‐digital levels qualified as independent prognostic markers of clinical endpoints.

Generally, a “gold standard paradox” comes into play when comparing visual and digital image analysis [41]. Digital analysis provides potentially more objective and reproducible datasets with a linear and continuous range of values. However, appraisal of specimen by experienced experimenters remains the current gold standard in pathology to validate results from digital analyses. From the data collected and taking into account reported correlations of EpCAM and Slug with clinical endpoints, the assessment of the membrane protein EpCAM was more precisely performed by the visual method, whereas Slug that is homogeneously distributed within the nucleus was more accurately defined by digital approaches. It must, however, be noted that it cannot be ruled out that an extended sample size might also result in significant stratification following digital assessment of EpCAM and visual assessment of Slug. Numerous parameters may account for the observed differences including differing area ratios of membranous versus nuclear localization and a potentially enhanced reliability in the definition of the nucleus as a more homogeneous geometric shape as opposed to cell membranes. Therefore, the definition of algorithms for the recognition of regions of interest in the context of automated software‐driven quantification solutions may represent a limitation that requires particular attention. Furthermore, human expertise, which is an intrinsic factor of visual quantification, reflects a higher degree of complexity of quantification than pure intensity scores.

## 5 Conclusion

HNSCC patients with pEMT features as measured in their primary tumors are more prone to develop metastases [9, 10]. Therefore, pEMT markers may serve as high‐risk parameters in addition to clinical parameters such as extra‐nodal extension of lymph node metastases and/or microscopically residual disease following surgical treatment. Likewise, pEMT markers might help in decision‐making for patients with comparable clinical parameters with respect to potential de‐escalation programs aiming at minimizing side effects of radio(chemo‐)therapy. Furthermore, the efficacy of EGFR‐targeted therapeutics in EGFR^high^/EpCAM^low^ carcinomas showing high Slug expression and high ERK1/2 phosphorylation levels [11] may profit from re‐evaluation. To address these requirements, we have performed a technical comparison of manual and optical scoring systems for two markers of pEMT in HNSCC samples. Digital quantification of Slug as marker for mesenchymal differentiation is feasible and represents a valuable objective tool with predefined criteria toward a fully digital assessment of pEMT as a surrogate for intratumor heterogeneity of HNSCC patients. Assessment of membrane protein EpCAM remained clinically more accurate upon visual grading. Fully digital assessment of pEMT in HNSCC may eventually serve as an additional stratification means in therapy decision‐making in the future.

## Conflict of interest

The authors declare no conflict of interest.

## Author contributions

Henrik Schinke performed all statistical analysis with the support of Daniel Samaga. Henrik Schinke, Timm Herkommer performed visual analyses. Theresa Heider, Timm Herkommer, Laura Dajka, Annette Feuchtinger, Axel Walch performed digital analyses and generated tissue microarrays. Laura Valeanu, Christoph Walz, Thomas Kirchner performed pathological appraisal of all samples within the study. Martin Canis, Philipp Baumeister, Claus Belka, Cornelius Maihöfer, Sebastian Marschner, Ulrike Pflugradt, Florian Simon, Ute Ganswindt, Julia Hess enrolled patient, build and curated the cohort used in the study. Gisela Kranz, Alexandra Blancke Soares performed immunofluorescence staining and confocal laser‐scanning microscopy for revisions. Horst Zitzelsberger and Olivier Gires designed and supervised the study and wrote the manuscript with the help of HS, TH, and JH.

## 6 Ethics approval and consent to participate

Clinical samples were collected based on written informed consent during routine surgery based on the approval by the ethics committee of the local medical faculties (Ethikkomission der Medizinischen Fakultät der Ludwig‐Maximilians‐Universität; EA 448‐13 and 17‐116) and in compliance with the WMA Declaration of Helsinki and the Department of Health and Human Services Belmont Report.

## Supporting information


**Fig. S1.** Digital and visual quantification of EpCAM and Slug. Absolute IHC score values (0‐300) from digital and visual quantification of EpCAM and Slug expression are represented with connecting lines and mean values (line) for each quantification method. Ns: not significant; ** p‐value < 0.01 Wilcoxon test.

**Fig. S2.** Univariate analysis for clinical endpoints. Univariable analyses of overall survival (OS), recurrence‐free survival (RFS), locoregional recurrence‐free survival (LR‐RFS), and disease‐specific survival (DSS). Shown are results from univariable Cox proportional hazard models including hazard ratios, 95% confidence intervals, Wald statistic p‐values, and significance levels (0.1 – 0.05; * 0.05; ** 0.01) for the variables T‐Status (tumor size), N‐Status (lymph node metastases), R‐Status (resection margin), Pn‐Status (perineural invasion), grading, ECE (extracapsular extension), HPV (human Papillomavirus), EpCAM and Slug quantification visual (Vis) and digital (Dig).

## Data Availability

Not applicable.
