# Supplementary figures and images for: Digital scoring of EpCAM and slug expression as prognostic markers in head and neck squamous cell carcinomas
Source: Mol Oncol. 2020 Dec 29;15(4):1040–53. doi: 10.1002/1878-0261.12886 (PMC8024715; doi:10.1002/1878-0261.12886)

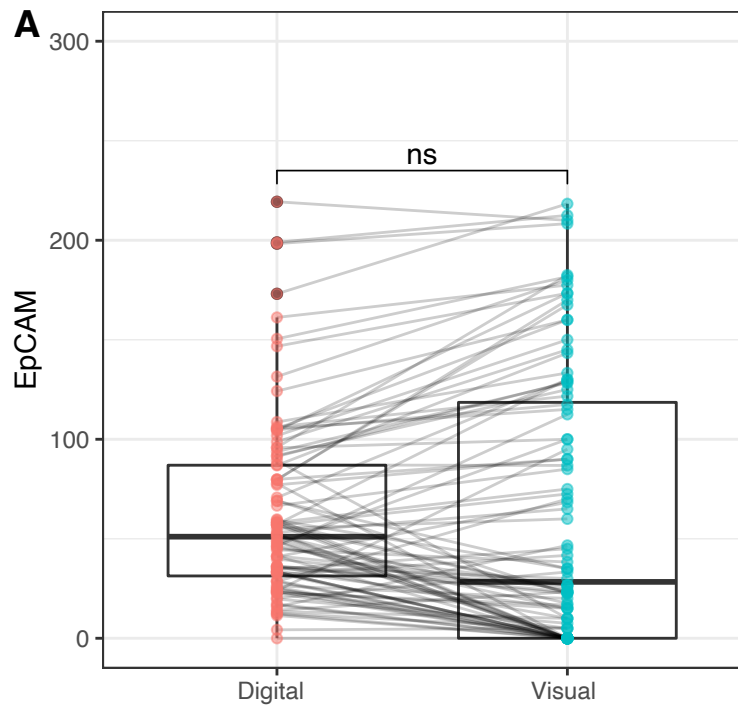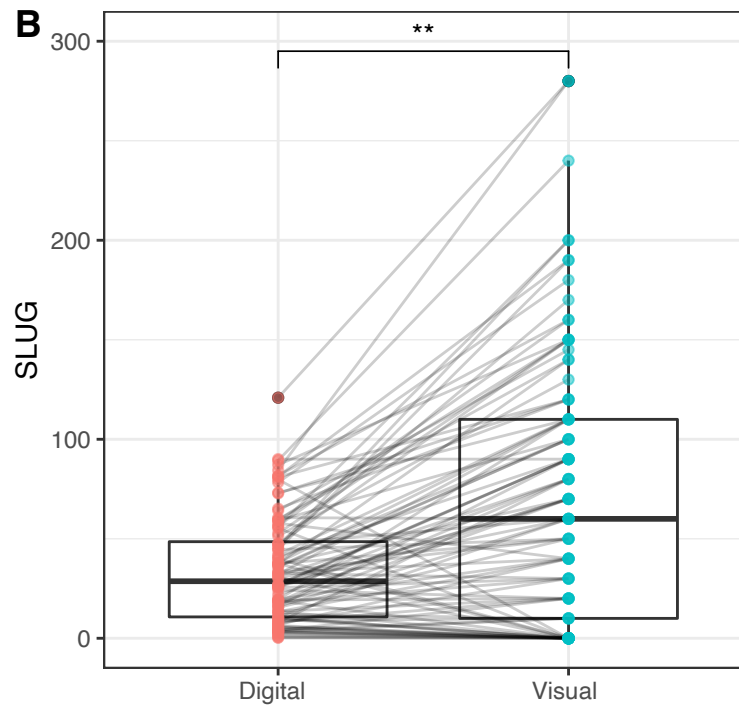

**Supplementary Figure 1**

Supplement: Supplementary file 1 — Fig. S1. Digital and visual quantification of EpCAM and Slug. Absolute IHC score values (0‐300) from digital and visual quantification of EpCAM and Slug expression are represented with connecting lines and mean values (line) for each quantification method. Ns: not significant; ** p‐value < 0.01 Wilcoxon test. [file MOL2-15-1040-s001.pdf]
