# Supplementary material for: Digital scoring of EpCAM and slug expression as prognostic markers in head and neck squamous cell carcinomas
Source: Mol Oncol. 2020 Dec 29;15(4):1040–53. doi: 10.1002/1878-0261.12886 (PMC8024715; doi:10.1002/1878-0261.12886)
Supplement: Supplementary file 2 — Fig. S2. Univariate analysis for clinical endpoints. Univariable analyses of overall survival (OS), recurrence‐free survival (RFS), locoregional recurrence‐free survival (LR‐RFS), and disease‐specific survival (DSS). Shown are results from univariable Cox proportional hazard models including hazard ratios, 95% confidence intervals, Wald statistic p‐values, and significance levels (0.1 – 0.05; * 0.05; ** 0.01) for the variables T‐Status (tumor size), N‐Status (lymph node metastases), R‐Status (resection margin), Pn‐Status (perineural invasion), grading, ECE (extracapsular extension), HPV (human Papillomavirus), EpCAM and Slug quantification visual (Vis) and digital (Dig). [file MOL2-15-1040-s002.pdf]

OS

ClinPara	ID	HR	Lower_CI	Upper_CI	p	signif
T-Status	2	0,696704	0,22440664	2,16302249	0,531822	
T-Status	3	1,140293	0,36742873	3,53883147	0,820263	
T-Status	4	2,790001	0,88269394	8,81857528	0,080558	.
N-Status	1	0,699038	0,25399753	1,92385529	0,488195	
N-Status	2	1,667912	0,78324822	3,55178685	0,184678	
N-Status	3	4,097342	0,87953745	19,0875452	0,072421	.
R-Status	1	0,579845	0,2558164	1,31430335	0,191774	
R-Status	NA	3,34E-08	0	Inf	0,99654	
Pn-Status	1	1,528731	0,71950258	3,24810441	0,269667	
Pn-Status	NA	0,682753	0,30205828	1,54325074	0,359053	
L-Status	1	2,675225	1,35600955	5,27786025	0,004534	**
L-Status	NA	1,940487	0,66440317	5,66747579	0,225402	
Grading	2	1,136408	0,15120112	8,54109389	0,901113	
Grading	3	0,677924	0,09063739	5,07054558	0,704959	
ECE	yes	1,953042	0,87356517	4,3664414	0,102959	
ECE	NA	1,28E-07	0	Inf	0,996879	
ECE	no	1,032937	0,4585963	2,3265761	0,937652	
HPV	pos	0,254618	0,07832947	0,82766358	0,022939	*
EpCAM.Dig		0,784439	0,51572849	1,19315536	0,256527	
SLUG.Dig		2,048215	1,18304142	3,54610168	0,010462	*
EpCAM.Vis		0,709456	0,53244721	0,94531103	0,019077	*
SLUG.Vis		1,142561	0,91016386	1,43429739	0,250696	

RFS

ClinPara	ID	HR	Lower_CI	Upper_CI	p	signif
T-Status	2	0,775766	0,25255155	2,38292798	0,657447	
T-Status	3	1,192831	0,384314	3,70229796	0,760266	
T-Status	4	3,247779	1,0388199	10,1538979	0,042821	*
N-Status	1	0,748191	0,2899395	1,93071182	0,548655	
N-Status	2	1,506382	0,72396745	3,13437817	0,273104	
N-Status	3	3,488718	0,75892079	16,0374451	0,10838	
R-Status	1	0,627604	0,28979445	1,35919414	0,237378	
R-Status	NA	3,4E-08	0	Inf	0,996454	
Pn-Status	1	1,491205	0,70178089	3,16864392	0,298769	
Pn-Status	NA	0,875013	0,4116258	1,86005629	0,728584	
L-Status	1	2,728058	1,38298475	5,38133284	0,003786	**
L-Status	NA	3,076473	1,23565972	7,6596227	0,015751	*
Grading	2	1,122412	0,14940717	8,43204232	0,910634	
Grading	3	0,743231	0,09986086	5,53161561	0,771999	
ECE	yes	1,812144	0,82806969	3,96568985	0,136796	
ECE	NA	1,23E-07	0	Inf	0,996726	
ECE	no	0,993574	0,45600529	2,16486325	0,987055	
HPV	pos	0,236826	0,07302067	0,76809339	0,016418	*
EpCAM.Dig		0,907137	0,61606842	1,33572449	0,621534	
SLUG.Dig		1,99339	1,13594061	3,49807412	0,016209	*
EpCAM.Vis		0,796501	0,61080886	1,03864628	0,09296	.
SLUG.Vis		1,129405	0,89885437	1,41909087	0,296205	

LR-RFS

ClinPara	ID	HR	Lower_CI	Upper_CI	p	signif
T-Status	2	0,757716	0,246658	2,3276519	0,628014	
T-Status	3	1,144133	0,3684944	3,5524053	0,815816	
T-Status	4	3,135018	1,0014215	9,8143861	0,049716	*
N-Status	1	0,76333	0,2958298	1,9696208	0,576566	
N-Status	2	1,525038	0,7331309	3,1723388	0,258779	
N-Status	3	3,86218	0,8386003	17,787299	0,082905	.
R-Status	1	0,640296	0,2956581	1,3866641	0,258138	
R-Status	NA	3,42E-08	0	Inf	0,996444	
Pn-Status	1	1,50409	0,7078968	3,1957845	0,288439	
Pn-Status	NA	0,880068	0,4139057	1,8712464	0,739939	
L-Status	1	2,763916	1,4014938	5,4507791	0,003345	**
L-Status	NA	3,032241	1,2195296	7,5393704	0,016985	*
Grading	2	1,095764	0,1458449	8,2327117	0,929177	
Grading	3	0,709193	0,0952679	5,279367	0,737246	
ECE	yes	1,854338	0,847595	4,0568553	0,122105	
ECE	NA	1,25E-07	0	Inf	0,996725	
ECE	no	1,008232	0,4628084	2,1964437	0,983535	
HPV	pos	0,238122	0,0734274	0,7722168	0,016821	*
EpCAM.Dig		0,87978	0,5976759	1,2950389	0,516139	
SLUG.Dig		2,048435	1,1646972	3,6027264	0,012803	*
EpCAM.Vis		0,782027	0,6008723	1,0177962	0,067436	.
SLUG.Vis		1,137296	0,9041697	1,4305293	0,271665	

DSS

ClinPara	ID	HR	Lower_CI	Upper_CI	p	signif
T-Status	2	0,576713	0,1117361	2,9766415	0,51098	
T-Status	3	1,11578	0,2251017	5,530677	0,893294	
T-Status	4	2,158085	0,4180329	11,141066	0,358355	
N-Status	1	0,468362	0,0908444	2,4147144	0,364699	
N-Status	2	1,490183	0,5088249	4,3642602	0,466867	
N-Status	3	3,030394	0,351454	26,129414	0,313144	
R-Status	1	0,338447	0,0777962	1,4723926	0,148677	
R-Status	NA	3E-08	0	Inf	0,997756	
Pn-Status	1	1,402121	0,487002	4,0368287	0,531028	
Pn-Status	NA	0,330965	0,0733484	1,4933936	0,15035	
L-Status	1	2,610121	0,9908755	6,8754651	0,052208	.
L-Status	NA	0,978437	0,1251521	7,6494005	0,983424	
Grading	2	0,622325	0,0790882	4,8969113	0,652258	
Grading	3	0,231822	0,0282572	1,9018647	0,173415	
ECE	yes	2,169349	0,7259826	6,4823505	0,165564	
ECE	NA	1,01E-07	0	Inf	0,998128	
ECE	no	0,574912	0,1543384	2,1415497	0,409378	
HPV	pos	3,57E-09	0	Inf	0,997791	
EpCAM.Dig		1,120209	0,6789113	1,848354	0,656841	
SLUG.Dig		2,793586	1,3060819	5,9752162	0,008089	**
EpCAM.Vis		0,860351	0,591791	1,2507871	0,430779	
SLUG.Vis		1,202933	0,8778413	1,648415	0,250382	

Supplementary Figure 2
